# Association between Interleukin-10 Gene Promoter Haplotype and Schizophrenia in a Han-Chinese Study

**Published:** 2008-09

**Authors:** Hsin-Yi Peng, Yu-Chi Ku, Bih-Ching Shu, For-Wey Lung

**Affiliations:** 1 *Department of Psychiatry, Kaohsiung Armed Forces General Hospital, Kaohsiung, Taiwan, ROC;*; 2 *Institute of Allied Health Sciences and Department of Nursing, National Cheng Kung University, Tainan, Taiwan, ROC;*; 3 *Graduate Institute of Behavioral Sciences, Kaohsiung Medical University, Taiwan, ROC;*; 4 *Department of Psychiatry, National Defense Medical Center, Taipei, Taiwan, ROC;*; 5 *Calo Psychiatric Center, Pingtung County, Taiwan, ROC*

**Keywords:** schizophrenia, *IL-10* promoter region, haplotypic analysis, community

## Abstract

Schizophrenia is a multi-factorial genetic disease, and it is caused by a combination of different gene polymorphisms and not individual ones, however, its pathogenesis is still unclear. The purpose of this study was explored the association between the *-1082G/A*, *-819T/C*, and *-592C/A* polymorphisms of interleukin-10 (*IL-10*) in schizophrenia. A total of 659 schizophrenics were recruited from a teaching hospital, whereas 411 healthy non-schizophrenic individuals were recruited from community in the same geographical area. The *-1082G/A*, *-819T/C* and *-592C/A* polymorphisms were genotyped by using PCR-RFLP, direct sequencing and TaqMan^®^ SNP assay. Both maximum likelihood method of UNPHASED program and Bayesian method of PHASE software were utilized for haplotypic analysis. An allelic frequency difference was found between the schizophrenics and community controls at *-1082G/A* polymorphism of *IL-10* promoter (χ^2^ =4.678, *P*=0.031). A haplotype of ACA was observed to be associated with schizophrenia after performing UNPHASED, PHASE and multivariate logistic regression analysis (*P*<0.001; *P*=0.001). In addition, the persons who carry haplotype ACA of *IL-10* promoter SNPs were estimated for 5.789 fold higher risk to develop schizophrenia than controls. We postulated this haplotype association might due to variant-specific effect on *IL-10* gene regulation, which leads to imbalance secretion of Th_1_/Th_2_ cytokines. Nevertheless, more detailed mechanism needs to be elucidated in further investigations in order to confirm this hypothesis.

## INTRODUCTION

The prevalence of schizophrenia is estimated 1% worldwide in the report of the World Health Organization in 2001, and 1-3% in Taiwan (1). Schizophrenia affects not only the personal life of the patient, but also the daily life of their family and social harmony.

The causes and risk factors of schizophrenia are still uncertain. According to previous epidemiological studies, several causes have been indicated that might be the vulnerable factors of schizophrenia, such as: mothers infected with the influenza virus during pregnancy (2), a child born in the winter (3, 4), mother had co-morbid illness at childbirth (5), exposure to toxoplasmosis in childhood (4), the mother was affected by major stress events during pregnancy (6), and so on. In addition to the above causes, family history, substance abuse, and neglect during childhood or the teenage period may are associated with schizophrenia (7-9). Other than socio-psycho-physiological factors, the dopamine D4 receptor (*DRD4*) gene was found to be associated with schizophrenia, which serve as an important genetic component confer to disease susceptibility (10, 11). More recently, the imbalanced cytokine secretion was suggested that involve in the pathogenesis of schizophrenia base on the general findings of immune abnormality of schizophrenics (12). Schwarz and colleagues have developed three hypotheses to explain why the immunity associates with schizophrenia. In their hypothesis, it was suggested infection, autoimmunity, and Th1/Th2 imbalancing are play essential role in the development of schizophrenia (13). Actually, there is no further evidence to prove any specific virus or parasite infection would result in schizophrenia. Only a few studies have addressed mothers who exposed to influenza virus type A, measles virus or Herpes simplex virus- type 1 during pregnancy would increase the risk of their children becoming schizophrenic (14). Eaton et al. reported 5 out of a listed 29 autoimmune illnesses had a higher prevalence in schizophrenics than in control subjects, and concluded schizophrenia might be associated with a large range of autoimmune disorders (15). However, autoimmune illnesses are relatively rare in schizophrenics and the contradictory finding also concludes a negative correlation between schizophrenia and autoimmune disease in a case-control study of rheumatoid arthritis (16). Th1/Th2 imbalance was first described for an association with schizophrenia in a cross sectional study, which recruited a subgroup of schizophrenics with negative symptoms and poor therapeutic outcome. In these patients, Th1 cell secretion was observed that shift to Th2-like cell immune reactivity (13). Furthermore, paranoid patients were observed for less Interleukin-10 (*IL-10*) production than non-paranoid and normal subjects, which indicating a decreased activity of cellular immunity (17, 18).

*IL-10* is a renowned cytokine synthesis inhibiting factor (CSIF), and is about 8-kDa in size. It is secreted by a variety of cells, including monocytes/macrophages, T cells, B cells and mast cells. In the central nervous system, *IL-10* is secreted by microglia and astrocytes (19-21). *IL-10* is a well-known cytokine that responsible for various cellular functions, two of these functions are dominantly inhibit the production of cytokine in macrophages, such as *TNF*, *IL-1*, chemokine and *IL-12*, and inhibit in an auxiliary manner macrophage activation when T-cells are activated (22). *IL-10* is consisted of 5 exons, and 4 introns, and is mapped on chromosome 1q31-q32 (23-25). Up-to-date, *-1082G/A*, *819 T/C* and *-592 C/A* are described as three major susceptible SNPs within the promoter region of *IL-10*; -1082G was also characterized as a higher *IL-10*-producing allele, which is located within an *ETS*-like binding site. In addition, the putative *ETS*-like protein was speculated that to be associated with inflammatory bowel disease (24). The *IL-10* protein production is significantly increased in controls who carry *-1082G* allele when compare to those with *-1082A* allele (26). Additionally, haplotype GCC of the three *IL-10* promoter SNPs was also identified for higher secretion of *IL-10* (27, 28). Allelic frequency of three promoter SNPs was vary greatly in different ethnic groups. According to previous reports, the *-1082G/A* was identified as most susceptible locus, which associate with schizophrenia in Caucasian populations (29), whereas *-592C/A* was identified as the most statistically-significant SNP in Chinese ethnic (30). Haplotype GCC of three *IL-10* promoter SNPs was firstly identified for a significantly association with schizophrenia in Caucasians (29), then similar results also replicated in Chinese cohort (31). Other than haplotype GCC, haplotype GTA was also reported to be associated with schizophrenia in Chinese population (30). It seems that *IL-10* might correlate to schizophrenia base on the hypotheses of Th2-like immunity shift of susceptible allele carrier.

In fact, gene–gene interactions seem more important than the contribution of an independent susceptibility gene to common human disease (33). It also believed that a single gene polymorphism is insufficient for precipitating schizophrenia (33). In this regard, we attempt to seek for the in-depth gene-gene interactions which might confer to the risk modulation of schizophrenia in current study. Hence, the first purpose of the present study was to explore the role of the *-1082G/A*, *-819T/C*, and *-592C/A* polymorphisms of *IL-10* in schizophrenia. Second, the interaction between DRD4 and the *IL-10* polymorphism in schizophrenia was further investigated.

## METHODS

### Participants

Participants who had been diagnosed with schizophrenia base on the criteria of the DSM-IV were recruited from the Psychiatric Department of Kaoshiung Armed Forces General Hospital. Six hundred and fifty-nine schizophrenics were recruited as subjects; of these 659 participants, 430 are male (69.80%), the average age is 36.14 years old (SD=11.4). A total of 411 controls with an average age of 45.17 (SD=13.7) were recruited and 178 (43.30%) were male. All controls were randomly selected from the same geographical area as the subjects who resident in a community of Southern Taiwan. They have confirmed as non-schizophrenic who diagnosed by at least two psychiatrists. Informed consents were obtained from all participants before data collection and blood sampling for genetic studies; as well the research protocol was approved by the Institutional Review Board of Kaohsiung Armed Forces General Hospital.

### DNA extraction from blood

The genomic DNA of all participants was prepared from 0.5 ml peripheral blood by using the NucleoSpin blood kit (Macherey-Nagel, Germany) following the manufacturer’s recommendations, then stored at -20°C freezer for further use.

### Genotyping of IL-10 *-1082G/A, -819T/C* and *-592C/A* promoter polymorphisms

TaqMan^®^ SNP Assay was used for allelic discrimination of *-1082G/A* polymorphism, which performed on a BIO-RAD IQ™5 multicolor real-time PCR detection system. Briefly, the PCR reaction were handled with a total of 25 ul volume containing 12.5 ul of 2X TaqMan Universal PCR Master Mix, 1.25 ul of each 20 uM primer and TaqMan probe, which labeled with FAM or VIC fluorescent dye and 9.25 ul of DNase-free water. The PCR condition is initially denatured at 95°C for 10 minutes, then 40 cycles at 92°C, 15 seconds for denaturation, and 60°C, 1 minute for annealing and extension. The probe sequence is 5'-TCC TCT TAC CTA TCC CTA CTT CCC C[T/C]T CCC AAA GAA GCC TTA GTA GTG TTG-3'. The *-819T/C* genotyping was done by using Polymerase Chain Reaction (PCR) and direct sequencing. PCR was firstly carried out in 25 ul volume reaction containing 50 mM KCl, 10 mM HCl, 0.5 mM MgCl_2_, 200 uM of dATP, dCTP, dTTP, 150 uM dGTP and 0.5 U Taq polymerase (Protech, Taipei, Taiwan). The thermal cycling condition is denaturation at 95°C for 5 min; follow 38 cycles of 94°C for 30 sec, 54°C for 45 sec, 72°C for 1 min, and 7 min for final extension at 72°C. Thereafter, the amplified fragment was subjected for cycle sequencing reaction, which performed by using BigDye terminator cycle sequencing kit version 3.1 according the manufacturer’s instructions. The extension products were separated on ABI 310 automatic DNA sequencer (Applied Biosystems, Foster City, CA) after completed BigDye cycle sequencing termination reaction. The sequencing primer was 5'-TTC AAC TTC TTC CAC CCC ATC-3' (forward primer) and 5'-GGC TCC TTT ACC CCG ATT TC-3' (reverse primer). *-592C/A* polymorphism was genotyped using polymerase chain reaction-restriction fragment length polymorphism (PCR-RFLP). In a few words, PCR was conducted in a 20 ul total volume with 50 mM KCl, 10 mM HCl, 0.5 mM MgCl_2_, 200 uM of dATP, dCTP, dTTP; 150 uM dGTP and 0.5U Taq polymerase (Protech, Taipei, Taiwan). The thermal cycling condition is denaturation at 95°C for 5 min, followed elongation by 38 cycles at 94°C for 30 sec; 56°C for 30 sec; 72°C for 1 minute, and 10 minutes at 72°C for final extension. The primers are 5'-GGT CAT GGT GAG CAC TAC CT-3' (forward primer) and 5'-AAA AAG TTG ATT TCC TGG GG-3' (reverse primer). The PCR products were subjected to restriction enzyme digestion with *RsaI* at 37°C for 4 hrs, and then separated on 3% agarose gel containing 0.5ug/ml ethidium bromide, finally visualized under a UV transilluminator. The PCR product of C allele homozygote was 493 bp in length, the C/A heterozygote was digested into three bands of 493 bp, 311 bp, and 182 bp, and the homozygote of the A allele was digested into 311 bp, and 182 bp DNA fragments.

### Statistical analysis

Allelic frequency of the *IL-10* promoter SNPs and demographic data were analyzed using chi-square test within SPSS package software for Windows, version 15.0. The maximum-likelihood method of UNPHASED program (version 3.0.4) (34, 35), which available on the following website: http://www.mrc-bsu.cam.ac.uk/personal/frank/software/unphased/ was utilized to calculate the EM algorithm in order to determine whether haplotype is statistically-significant associate with schizophrenia. In addition, the Bayesian method of PHASE program version 2.1, which available on (http://www.stat.washington.edu/stephens/software.html) was applied to reconstruct and confirm haplotype achieved previously from UNPHASED analysis by processed the genetic information of the subjects vs. controls. The information of *IL-10* haplotype distribution, age, gender, and *DRD4* exon3 VNTR polymorphism were converted into dummy variables and brought to be analyzed in a multivariate logistic regression model in order to explore the significance of these factors, relative odd ratio, and possible gene-gene interaction.

## RESULTS

### Allele frequencies and genotypes of *IL-10 -1082G/A, -819T/C* and *-592C/A* promoter polymorphisms

A statistically-significant difference was found for the allelic frequency distribution of *IL-10 -1082G/A* polymorphism between subjects and controls (χ^2^=4.703, *df*=1, *P*=0.031, OR=1.6984), which as shown in Table 1. The A allele of *-1082G/A* polymorphism was observed more frequently in schizophrenics, which indicating it might be a risk allele that associate with disease vulnerability. In addition, the genotypic distribution of *-1082G/A* polymorphism also be found for a statistically-significant difference between two subgroups (χ^2^=7.242, *df*=2, *P*=0.027, which as shown in Table 2). No statistically-significant difference was found for *-819T/C* polymorphsim, but homozygote of C allele observed more frequent in subject group (11.09%) than in the control group (10.46%). It was also fail to find an association between *-592C/A* polymorphism and schizophrenia; however, A allele homozygote was also frequently detected in the subject group (50.99%) when compare to control group (49.64%) (Table 2).

**Table 1:** Allele frequency of *IL-10 -1082G/A, -819T/C* and *-592C/A* polymorphisms in schizophrenia and community groups

	Schizophrenia	Community	OR	*p* value	χ^2^

Genotype	n= 1318 (%)	n= 822 (%)			
IL-10 -1082					
A	712 (96.48%)	774 (94.16%)	1.6984	0.031^a^	4.678
G	26 (3.52%)	48 (5.834%)	1	(df=1)	
IL-10 -819					
C	366 (29.00%)	242 (29.44%)	1	0.8377	0.042
T	896 (71.00%)	580 (70.56%)	1.021	(df=1)	
IL-10 -592					
A	947 (71.85%)	574 (69.83%)	1.103	0.3208	0.9855
C	371 (28.15%)	248 (30.17%)	1	(df=1)	

a *P*<0.05.

**Table 2:** Genotype and Hardy-Weinberg analysis of *IL-10 -1082G/A, -819T/C* and *-592C/A* polymorphisms in schizophrenia and community groups

	Schizophrenia	Community	*p* value	χ^2^

Genotype	n=659 (%)	n=411 (%)		
IL-10 -1082				
AA	345 (93.50%)	364 (88.60%)	0.027^a^	7.242
AG	22 (5.60%)	46 (11.08%)	(df=2)	
GG	2 (0.54%)	1 (0.24%)		
HWE *p*	0.061	0.937		
IL-10 -819				
CC	70 (11.09%)	43 (10.46%)	0.775	0.510
CT	226 (35.82%)	156 (37.96%)	(df=2)	
TT	335 (53.09%)	212 (51.58%)		
HWE *p*	0.005^b^	0.216		
IL-10 -592				
AA	336 (50.99%)	204 (49.64%)	0.307	2.363
AC	275 (41.73%)	166 (40.39%)	(df=2)	
CC	48 (7.28%)	41 (9.98%)		
HWE *p*	0.719	0.703		

a *P*<0.05;

b *P*<0.01.

### Haplotype Association between *IL-10* promoter SNPs and schizophrenia

Eight possible 3-marker haplotypes were constructed after performing haplotypic analysis by using maximum likelihood method of UNPHASED program. These 3-marker haplotypes were haplotype ATA, ATC, GTA, GTC, ACA, ACC, GCA, and GCC. Among these haplotypes, haplotype GTC and GCA were excluded due to extremely low frequency, whereas haplotype ACA was the most significant one, which associated with disease vulnerability of schizophrenia as shown in Table 3 (χ^2^=12.000, OR=5.854, *P*<0.001). Moreover, the individuals who carry haplotype ACA were estimated for 5.854 fold higher risk to develop schizophrenia when compare to the non-carrier. This finding was consistent to previous allelic and genotypic results of three *IL-10* promoter SNPs, it was suggested that *-1082A* probably the major contributor of the haplotype association. The allelic information and genotypic data were further analyzed using PHASE program and haplotype ACA had again been identified for its significantly association with schizophrenia. In addition, multivariate logistic regression also showed that haplotype ACA statistically-significant associate with schizophrenia (*P*=0.001) after controlling the variables of sex, age and *DRD4* exon3 VNTR polymorphism (Table 4). The haplotype ACA was identified for an association with higher vulnerability of schizophrenia (OR=5.789, *P*=0.001) in current study, but gene-gene interaction was not found.

**Table 3:** Haplotype analysis of *IL-10 -1082G/A, -819T/C* and *-592C/A* polymorphisms in schizophrenia and community groups, with the maximum likelihood method of UNPHASED program

	Schizophrenia frequency	Community frequency	OR	*p* value	χ^2^

Haplotype ACA	3.19	0.86	5.854	<0.001^a^	12.00
Haplotype ACC	22.93	23.19	1.570	0.9241	0.009
Haplotype ATA	68.78	68.51	1.594	0.9087	0.013
Haplotype ATC	1.55	1.60	1.542	0.9004	0.016
Haplotype GCC	3.39	5.38	1.000	0.1299	2.294
Haplotype GTA	0.16	0.46	0.565	0.3029	1.061

a *P*<0.01.

**Table 4:** Haplotype analysis of *IL-10 -1082G/A, -819T/C* and *-592C/A* polymorphisms in schizophrenia and community groups

	B	S.E.	Wald	df	*p* value	OR	95% CI for EXP (B)	
							lower	upper

**Haplotype ACA**	1.756	0.513	11.731	1	0.001^a^	5.789	2.119	15.812
**Gender**	-0.989	0.170	33.629	1	<0.001^a^	0.372	0.266	0.520
**Age**	-0.060	0.007	69.729	1	<0.001^a^	0.942	0.929	0.955
**DRD4**	0.894	0.448	3.974	1	0.046	2.445	1.015	5.888
**Constant**	1.292	0.294	71.902	1	<0.001^a^	3.641		

Bayesian methods for reconstructing haplotypes using PHASE and logistic regression.

a *P*<0.01.

## DISCUSSION

Our gene association study of a Taiwanese Han ethnic suggested that the *IL-10 -1082A* allele might be associated with schizophrenia (OR=1.6984, *P*=0.030), which contradict to the previous report of *-1082G* allele might associated with enhanced risk of schizophrenia in Caucasian population and another published work of Yu *et al* that analyzed a Chinese population sample set (29, 30). Among three promoter SNPs of *IL-10*, other than -1082G/A polymorphism, another work done by analyzed Chinese ethnic samples conclude that *-592A* allele was associated with schizophrenia as well (30). In current study, the homozygote of *-1082A* allele was found more frequently among schizophrenics, whereas *-1082G* homozygosity was reported previously for higher prevalence among the schizophrenics in western countries. It was suggested that ethnic difference might confound the genetic effect of *-1082G/A* polymorphism on disease risk modulation of schizophrenia. In this study, different statistical methodologies were utilized for genetic analysis. Both UNPASED and PHASE program confirmed the haplotype association between 3-marker haplotype ACA and schizophrenia. In addition, the statistically significance still remained after controlling the variables of age, gender and *DRD4* genotypic data in the multivariate logistic regression analysis. The statistical power was calculated using algorithms described elsewhere (38-40) for logistic regression with binary covariates, which aslo available on http://www.dartmouth.edu/~eugened/power-samplesize.php for quick access. Our result of haplotype association of 3-marker haplotype ACA to the schizophrenia was estimated for a 100% power when *P* value less than 0.05 was considered statistical significant.

In the previous work of Yu (30) and He (31), the studied cohorts were apparently recruited from the same ethnic group, but cross-ethnic marriages are common in China, a vast country with numerous ethnic groups, thus, representatives of a single ethnic origin might absent. Additionally, that the subjects were not randomly selected from a population would more easily lead to bias and result in misleading conclusions. Other than these factors, variations might occur across independent studies because of sample size, ethnic differences, and lack of controlling other environmental factors that associated with schizophrenia, such as being born in winter, the mother having the flu during pregnancy, the mother having co-morbid illnesses at childbirth, exposure to *Toxoplasma gondii* virus during childhood, the mother being effected by major stress events during pregnancy, family history, abuse or neglect in childhood or the teenage period, substance abuse, etc (5, 6, 14, 23).

The susceptible gene region of systemic lupus erythematosus (SLE), a common autoimmune disease, was mapped on chromosome 1q21-q44S, and *IL-10* was identified to be located within this region (36). Haplotype GCC of *IL-10* was found to be associatied with SLE in Caucasians (37); in addition, haplotype GCC also functional characterized for a higher *IL-10* expression (27, 28). Other than haplotype GCC, haplotype ACC was suggested that to be associated with SLE in a study of Thailand (36); thereby, again we saw that ethnic differences probably contribute to susceptibility to autoimmunity disease. Both schizophrenics and SLE patients reveal abnormalities in their immune secretion, which possibly due to similar mechanisms triggered by the same genetic component. *IL-10* promoter SNPs were speculated play an important role in *IL-10* gene regulation, which associate with critical immune responses in the early stage of brain development. Alternatively, *IL-10* expression probably triggers Th2 cell-derived immune responses to infectious pathogens or other environmental stressors in neonates; subsequently these cellular events might play a pivotal role in schizophrenia development. In addition, the different diagnostic subtypes of schizophrenia, paranoid or non-paranoid, also be found for an immunologically difference in *IL-10* response, which indicating a possibility that *IL-10* involved in the pathogenesis of schizophrenia (17, 18). Thus, we highly suspect that individual who carry haplotype ACA of *IL-10* promoter SNPs (*-1082A, -819C, -592A*) might be at higher risk to develop schizophrenia later in their life. According our preliminary findings, it was estimated for 5.8 fold higher risk in development of schizophrenia either through UNPHASED or PHASE analysis. The mechanism of this haplotype association might due to abnormal (probably less) secretion of *IL-10*, which response to various stressor toward a Th1/Th2 misbalancing. However, further studies are necessary to confirm these findings.
